# Exposure–safety–efficacy analysis of single-agent ixazomib, an oral proteasome inhibitor, in relapsed/refractory multiple myeloma: dose selection for a phase 3 maintenance study

**DOI:** 10.1007/s10637-016-0346-7

**Published:** 2016-04-02

**Authors:** Neeraj Gupta, Richard Labotka, Guohui Liu, Ai-Min Hui, Karthik Venkatakrishnan

**Affiliations:** Clinical Pharmacology, Millennium Pharmaceuticals, Inc., a wholly owned subsidiary of Takeda Pharmaceutical Company Limited, 40 Landsdowne Street, Cambridge, MA 02139 USA; Clinical Research, Millennium Pharmaceuticals, Inc., a wholly owned subsidiary of Takeda Pharmaceutical Company Limited, 40 Landsdowne Street, Cambridge, MA 02139 USA; Biostatistics, Millennium Pharmaceuticals, Inc., a wholly owned subsidiary of Takeda Pharmaceutical Company Limited, 40 Landsdowne Street, Cambridge, MA 02139 USA

**Keywords:** 20S proteasome, Ixazomib, Exposure–response, Maintenance, Multiple myeloma, Proteasome inhibitor

## Abstract

*Background* Ixazomib is the first oral, small molecule proteasome inhibitor to reach phase 3 trials. The current analysis characterized the exposure-safety and exposure-efficacy relationships of ixazomib in patients with relapsed/refractory multiple myeloma (MM) with a purpose of recommending an approach to ixazomib dosing for maintenance therapy. *Methods* Logistic regression was used to investigate relationships between ixazomib plasma exposure (area under the curve/day; derived from individual apparent clearance values from a published population pharmacokinetic analysis) and safety/efficacy outcomes (hematologic [grade ≥ 3 vs ≤ 2] or non-hematologic [grade ≥ 2 vs ≤ 1] adverse events [AEs], and clinical benefit [≥stable disease vs progressive disease]) using phase 1 data in relapsed/refractory MM (NCT00963820; *N* = 44). *Results* Significant relationships to ixazomib exposure were observed for five AEs (neutropenia, thrombocytopenia, rash, fatigue, and diarrhea) and clinical benefit (*p* < 0.05). Dose–response relationships indicated a favorable benefit/risk ratio at 3 mg and 4 mg weekly, which are below the maximum tolerated dose of 5.5 mg. At 3 mg, the model predicted that: 37 % of patients will achieve clinical benefit; incidence of grade ≥ 3 neutropenia and thrombocytopenia will be 10 % and 23 %, respectively; and incidence of grade ≥ 2 rash, fatigue, and diarrhea will be 8 %, 19 %, and 19 %, respectively. *Conclusions* Based on the findings, patients in the phase 3 maintenance trial will initiate ixazomib at a once-weekly dose of 3 mg, increasing to 4 mg if acceptable tolerability after 4 cycles, to provide maximum clinical benefit balanced with adequate tolerability.

## Introduction

The proteasome inhibitor ixazomib is the first oral, small molecule inhibitor of the 20S proteasome to be investigated in the clinic [1]. Following demonstration of preclinical activity against multiple myeloma (MM) cell lines and in-vivo models [2–5], ixazomib has demonstrated encouraging early-phase clinical activity with very high response rates (including high ≥very good partial response [VGPR] rates) and a manageable toxicity profile, with limited peripheral neuropathy, in single-agent use in relapsed/refractory MM [6, 7] and when given in combination with lenalidomide and dexamethasone or melphalan and prednisone in newly diagnosed multiple myeloma [8–11]. Ixazomib is now in phase 3 clinical development in relapsed and/or refractory MM, newly diagnosed MM, and relapsed/refractory primary systemic light chain (AL) amyloidosis. In two ongoing, randomized phase 3 trials of ixazomib in combination with lenalidomide and dexamethasone versus placebo plus lenalidomide and dexamethasone in newly diagnosed (TOURMALINE-MM2; clinicaltrials.gov identifier NCT01850524) and relapsed and/or refractory (TOURNALINE-MM1; NCT01564537) MM, patients are receiving an ixazomib dose of 4 mg weekly (one dose level below the maximum tolerated dose [MTD] of 5.5 mg determined in a previous phase 1/2 trial) [8]. In November, 2015, the United States (US) Food and Drug Administration (FDA) granted approval ixazomib for use (at a starting dose of 4 mg) in combination with lenalidomide and dexamethasone for the treatment of patients with MM who have received at least one prior therapy, based on results from TOURMALINE-MM1 [12, 13].

Despite extensive research in both the post-transplant and non-transplant settings (including with bortezomib) [14–25], to date, there are no drugs approved for maintenance therapy in MM. The balance of benefit to risk is paramount for maintenance therapy when patients already have a clinical response to high-dose therapy (HDT), are likely to be symptom-free from their disease, and have not had prior exposure to non-induction therapy agents before starting maintenance. Hence, any maintenance therapy should ideally have an acceptable tolerability profile, a low rate of discontinuations due to adverse events (AEs), simple and convenient administration, proven effectiveness (prolonged survival and improved quality of life [QoL]), and a favorable cost/benefit ratio. These considerations will be crucial in order to maximize patient adherence and maintenance of the anticancer effects during relatively long-term administration in the maintenance setting compared to settings of advanced disease [26].

A phase 3, randomized, placebo-controlled, double-blind study of oral ixazomib maintenance therapy in MM patients who have achieved at least partial response (PR) to induction therapy followed by HDT with autologous stem cell transplantation (HDT-ASCT) was recently initiated at the end of 2014 (NCT02181413). The primary goal of that trial is to determine the efficacy of single-agent ixazomib maintenance therapy. To select an appropriate dose for this maintenance study, we conducted exposure–response analyses of safety and efficacy data from patients enrolled in a phase 1 study of weekly single-agent ixazomib in relapsed and/or refractory MM (NCT00963820) [6]. This analysis was designed to yield initial estimates of a biologically active exposure/dose range of ixazomib associated with disease control and acceptable tolerability, thereby ensuring adequate tolerability for long-term treatment while maintaining drug exposures in the biologically active range.

## Methods

### Patients

The sample selected for these analyses comprised patients with relapsed and/or refractory MM enrolled in an open-label, phase 1, dose-escalation trial that was designed to evaluate safety and tolerability, to determine the MTD of single-agent ixazomib given on a once-weekly schedule for 3 out of 4 weeks, and to assess preliminary efficacy of ixazomib [6]. Oral ixazomib (dose range, 0.24–3.95 mg/m^2^) was administered to patients on an empty stomach [27] on days 1, 8, and 15 in 28-day cycles for up to 12 cycles, or until disease progression (PD) or unacceptable toxicity. However, patients could continue on therapy beyond 12 cycles if still deriving benefit based on the treating physician’s judgement. The study utilized a standard 3 + 3 dose-escalation design based on the presence of cycle 1 dose-limiting toxicities (DLTs). After determining the MTD, patients were enrolled into 1 of 4 expansion cohorts based on their relapsed/refractory status and prior exposure to other proteosome inhibitors. Study endpoints included safety and tolerability, MTD, pharmacokinetics (PK), and best anti-myeloma response.

All available data from patients for whom both PK and safety/efficacy information were available were included in the present analysis (*N* = 44). Data were available over a wide ixazomib dose range (0.48–3.95 mg/m^2^), corresponding to a fixed-dose range of approximately 0.8 to 8.9 mg [6, 28]. Within this dose range, the MTD of weekly oral ixazomib had been established as 2.97 mg/m^2^, equating to a fixed dose of 5.5 mg [6, 28].

### Pharmacokinetic analysis

A population PK model developed for ixazomib using data obtained from four phase 1 clinical studies was used for PK analysis and calculation of time-averaged exposure [28]. This three-compartment model with linear distribution and elimination kinetics and first-order linear absorption adequately describes the PK of ixazomib in plasma after intravenous or oral administration. The final model includes body surface area (BSA) as a covariate on the peripheral volume of the third compartment (V_4_). However, BSA was not identified as a covariate on ixazomib clearance (CL; which dictates total systemic exposure following fixed dosing). Therefore, BSA was concluded to not impact the total systemic exposure as characterized by the area under the curve (AUC) [28].

### Exposure–response analyses

The relationship between ixazomib exposure and safety/efficacy was analyzed using logistic regression. For both the exposure–safety and exposure–efficacy analyses, the metric of exposure was the time-averaged systemic exposure (AUC/day), calculated using the available dosing information for each patient, the individual patient oral clearance (CL/F) values estimated from the final population PK model, and the number of days to the first event. All logistic regression analyses were conducted using SPLUS software version 8.1 (TIBCO Software Inc., Palo Alto, CA, USA).

### Exposure–safety analyses

Exposure–safety analyses were conducted for selected hematologic (anemia, neutropenia, and thrombocytopenia) and non-hematologic (rash, fatigue, diarrhea, and peripheral neuropathy) AEs. AEs were included in the analysis if they occurred from the first day of ixazomib dosing until 30 days after the last dose of ixazomib. The dependent variable was the presence or absence of a given AE (≥grade 3 hematologic AEs; ≥grade 2 non-hematologic AEs) during the study. The intensity for each AE was determined according to the National Cancer Institute Common Toxicity Criteria for Adverse Events (NCI CTCAE) version 4.03. If the AE occurred more than once in an individual patient, the time to the first occurrence of the worst grade for the AE was used in the analysis.

To distinguish an AE profile that would be tolerable for long-term administration of a maintenance drug, hematologic AE data for anemia, neutropenia, and thrombocytopenia were categorized into grade ≥ 3 versus grade ≤ 2 groups (including those with no hematologic AE mentioned above), while non-hematologic AE data for rash, fatigue, diarrhea, and peripheral neuropathy were grouped into grade ≥ 2 versus grade ≤ 1 (including those with no non-hematologic AE mentioned above). The different cut-offs between hematologic and non-hematologic AE data were used because grade 2 hematologic AEs per se, defined based on laboratory test results alone (hemoglobin <10.0–8.0 g/dL, neutrophil count <1500–1000/mm^3^, platelet count <75,000–50,000/mm^3^) without associated clinical symptoms or sequelae would not be expected to have a clinically significant impact on QoL (which is important for maintenance therapy) and, in principle, should be more manageable than grade 2 non-hematologic AEs (e.g. diarrhea) which can be expected to be particularly detrimental to QoL and can adversely impact treatment adherence in the setting of long-term maintenance therapy [26, 29, 30].

### Exposure–efficacy analyses

Time-averaged ixazomib exposure to the time of the first confirmed best clinical response, to discontinuation from treatment due to any reason, or to the point of starting alternative therapy (whichever was first) was used for the exposure–efficacy analysis.

The analysis was based on PK- and response-evaluable patients in the exposure–safety–efficacy analysis population (*N* = 44). The exposure–efficacy relationship was characterized by logistic regression models relating ixazomib exposure to the probability of achieving a best response of stable disease or better (≥SD) as evaluated by the International Myeloma Working Group (IMWG) Criteria. Data for the exposure–efficacy analyses were categorized as ≥SD versus PD groups because the clinical benefit rate (including SD in patients with relapsed/refractory MM) was considered to be a clinically meaningful indicator of disease control, for purposes of translation to anti-myeloma activity in the maintenance setting.

## Results

### Patients

A total of 44 patients were included in the exposure–safety–efficacy analysis (Table 1); 55 % were male and the median age was 65 years (range, 40–79). Twenty-six patients (59 %) were treated with ixazomib at a dose level of ~2.97 mg/m^2^ and five patients (11 %) received 3.95 mg/m^2^. Across all patients included in the study (*N* = 60), 85 % of patients had received bortezomib and 15 % had received carfilzomib as prior therapy [6].

**Table 1:** Patient demographics

Category	All patients (*N* = 44)
Median age, years (range)	65 (40–79)
Male/female, n (%)	24 (55)/20 (45)
Race, n (%)	
White	39 (89)
Black	3 (7)
Other	2 (5)
Mean BSA, m^2^ (SD)	1.97 (0.24)
Ixazomib dose range,* mg	0.8–8.9
Ixazomib dose level, n (%)	
0.48 mg/m^2^	1 (2)
0.80 mg/m^2^	3 (7)
1.20 mg/m^2^	2 (5)
1.68 mg/m^2^	4 (9)
2.23 mg/m^2^	3 (7)
2.97 mg/m^2^	26 (59)
3.95 mg/m^2^	5 (11)

*Actual administered dose

*BSA* body surface area, *SD* standard deviation

### Logistic regression analyses

**Fig. 1:**
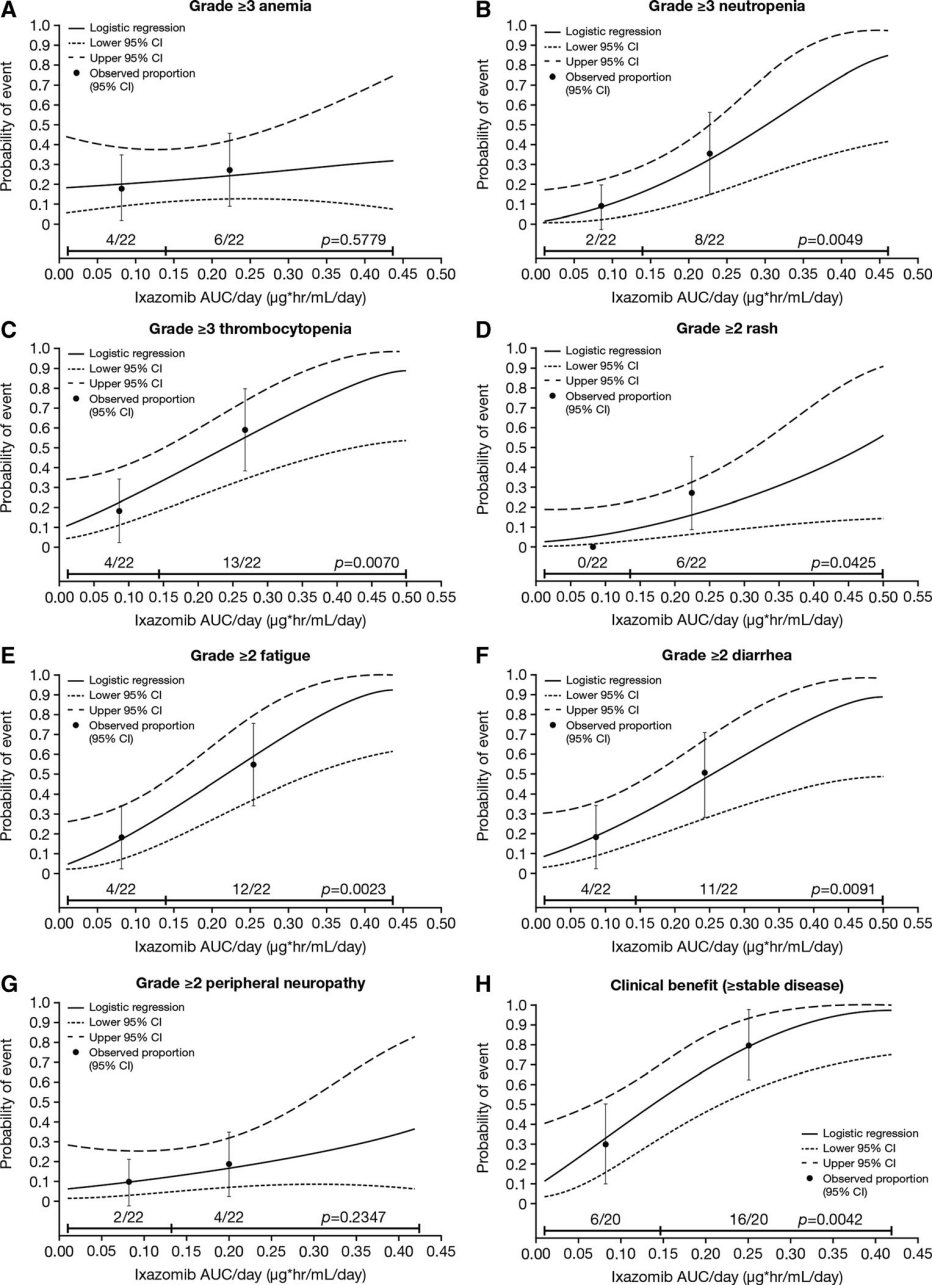
Ixazomib time-averaged exposure versus individual adverse events of clinical importance (grade ≥ 2 for non-hematologic and grade ≥ 3 for hematologic adverse events) and clinical benefit rate (≥stable disease) with single-agent weekly ixazomib (*N* = 44). Ixazomib exposure range in each group (below vs above median) is denoted by the *horizontal black line*. *Black dots* (*vertical lines*) represent the observed proportion of patients (95 % CI) in each group (below vs above median). n/N is the number of patients with events/total number of patients in each group (below vs above median). AUC, area under the plasma concentration–time curve; CI, confidence interval

The results from the logistic regression analyses indicated that, of the seven evaluated AEs, significant relationships to ixazomib exposure were observed for five of these AEs: neutropenia, thrombocytopenia, rash, fatigue, and diarrhea (all *p* < 0.05; Fig. 1); no such relationship was discernible for anemia and peripheral neuropathy. There was also a significant relationship between the clinical benefit rate (≥SD) and ixazomib exposure.

**Fig. 2:**
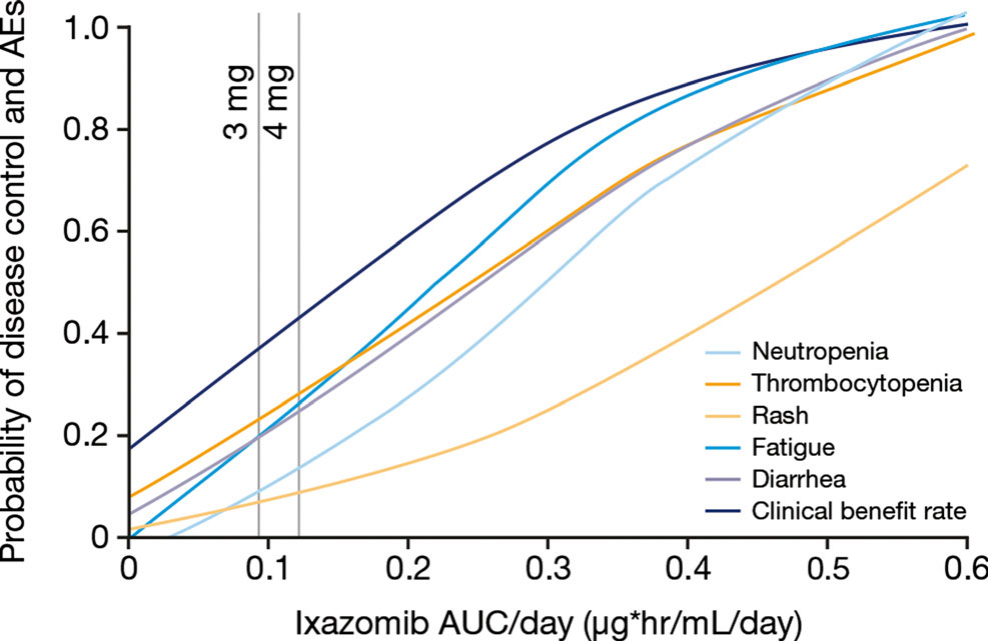
Relationships between adverse events (grade ≥ 3 for hematologic and grade ≥ 2 for non-hematologic adverse events) or clinical benefit rate (≥stable disease) with single-agent weekly ixazomib, and ixazomib exposure associated with 3 mg and 4 mg fixed doses (*N* = 44). AEs, adverse events; AUC, area under the plasma concentration–time curve

Dose-response relationships were inferred from the estimated exposure–response relationships, based on population mean time-averaged AUC values associated with different fixed doses (Fig. 2 and Table 2). These analyses indicated that a favorable benefit/risk ratio may be achieved at doses of 3 mg (55 % of the MTD) and 4 mg (73 % of the MTD), both of which are below the 5.5 mg MTD for weekly, single-agent ixazomib and were tested in the phase 1 safety and efficacy study in relapsed/refractory MM (NCT00963820) [6]. At a starting dose of ixazomib 3 mg weekly, the model predicted that: approximately 37 % of patients will achieve clinical benefit (≥SD); incidence of grade ≥ 2 rash, fatigue, and diarrhea will be 8 %, 19 %, and 19 %, respectively; and incidence of grade ≥ 3 neutropenia and thrombocytopenia will be 10 % and 23 %, respectively (Table 2). The model predictions for a 4 mg dose were as follows: approximately 44 % of patients will achieve clinical benefit (≥SD); incidence of grade ≥ 2 rash, fatigue, and diarrhea will be 10 %, 26 %, and 24 %, respectively; and incidence of grade ≥ 3 neutropenia and thrombocytopenia will be 16 % and 28 %, respectively.

**Table 2:** Probability of grade ≥ 3 hematologic adverse events, grade ≥ 2 non-hematologic adverse events, and clinical benefit at weekly ixazomib doses of 3 mg and 4 mg, as estimated from the logistic regression exposure-response analyses

	Estimated probability of event, %	
Outcome measure	Ixazomib 4 mg weekly	Ixazomib 3 mg weekly
Neutropenia, grade ≥ 3	16	10
Thrombocytopenia, grade ≥ 3	28	23
Rash, grade ≥ 2	10	8
Fatigue, grade ≥ 2	26	19
Diarrhea, grade ≥ 2	24	19
Clinical benefit rate (≥SD)	44	37

*SD* stable disease

## Discussion

We conducted exposure–response analyses using safety, efficacy, and PK data from a phase 1 trial of single-agent, weekly ixazomib in relapsed/refractory MM in order to determine an appropriate dose for a phase 3 randomized, double-blind maintenance study (NCT02181413). The phase 3 trial will aim to assess the efficacy and safety of ixazomib maintenance versus placebo in patients with newly diagnosed MM who have achieved ≥PR to prior proteasome inhibitor and/or immunomodulatory drug-based induction therapy followed by HDT-ASCT. The primary endpoint will be progression-free survival, with overall survival a key secondary endpoint.

Long-term treatment in settings like maintenance therapy of myeloma will require selection of doses that provide the right balance between offering bioactive exposures associated with disease control and a tolerable safety profile without toxicities that adversely impact QoL [26, 30]. Therefore, based on the findings from the present logistic regression analyses, it was decided that ixazomib maintenance therapy will be initiated at a once-weekly dose of 3 mg. At a weekly ixazomib dose of 3 mg, the analysis predicted that the probabilities of grade ≥ 3 hematologic and grade ≥ 2 non-hematologic AEs would be reduced compared to the 4 mg dose. Further, the 3 mg dose is within the clinically active range for ixazomib (as indicated by the relationship between ixazomib exposure and clinical benefit at this dose, where the probability of achieving clinical benefit [≥SD] was predicted to be 37 %). This represents one dose level below the starting dose (4 mg) used in ongoing phase 3 trials in relapsed/refractory (NCT01564537) [13] and previously untreated (NCT01850524) MM, and the dose that is indicated for use in combination with lenalidomide and dexamethasone for the treatment of patients with relapsed and/or refractory MM [12]. Notably, in three maintenance clinical trials of the immunomodulatory drug lenalidomide as maintenance therapy for MM, lenalidomide was also administered at a reduced starting maintenance dose (10 mg) [31–33], relative to the 25 mg dose that is recommended in the front-line or relapsed settings [34].

Various phase 1/2 trials have characterized the clinical pharmacology, safety and efficacy of ixazomib in MM [6–10]. Although most of these trials utilized body surface area (BSA)-based dosing, a recent analysis has shown that it is feasible to switch from BSA-based to fixed dosing of ixazomib with negligible effects on the PK characteristics of the drug [28]. As such, all ixazomib studies now employ a fixed-dosing approach and this is reflected in the United States Prescribing Information for ixazomib [12].

**Fig. 3:**
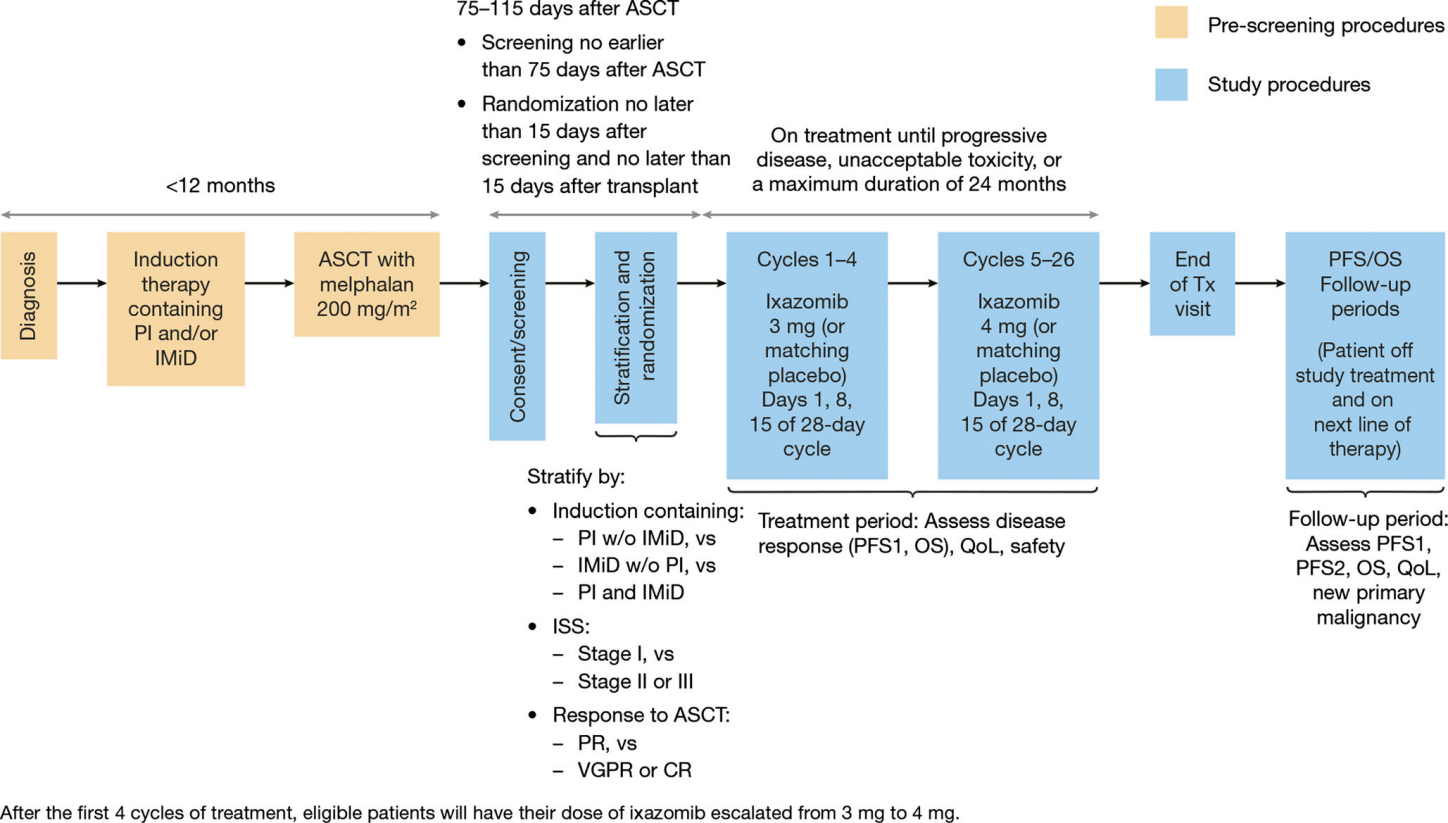
Design of the phase 3 study of ixazomib maintenance following induction therapy and high-dose therapy/autologous stem cell transplantation in newly diagnosed patients with multiple myeloma. ASCT, autologous stem cell transplantation; CR, complete response; IMiD, immunomodulatory drug; ISS, International Staging System; OS, overall survival; PFS, progression-free survival; PI, proteasome inhibitor; QoL, quality of life; Tx, treatment; VGPR, very good partial response; w/o, without

In addition to informing the starting dose used in phase 3 clinical trials, performing dose comparison assessments in early phase trials can be used to define dose-modification strategies in subsequent phase 3 trials [26, 35]. To provide patients in the phase 3 trial of ixazomib maintenance therapy the opportunity to derive maximum clinical benefit (without prohibitive toxicity), the starting dose of 3 mg will be increased to 4 mg after 4 cycles (i.e. at cycle 5, day 1) based on the observed tolerability at the 3 mg dose in individual patients. Such a posology was selected instead of starting treatment at the 4 mg dose in all patients in order to optimize the overall benefit/risk profile in the maintenance setting, where long-term therapy without excessive toxicity is an important consideration. While starting all patients at a 4 mg weekly dose is appropriate in the treatment setting due to the need to rapidly induce a clinical response, such an approach was not considered in the maintenance therapy setting as it may lead to a significant impact on QoL in the early stage of treatment, which may impact the long-term treatment adherence, and even result in early discontinuation. The ixazomib dose will only be increased if, during cycles 3 and 4, there have been no drug-related non-hematologic grade ≥ 2 AEs, no drug-related dose interruptions, and no drug-related delays of >1 week in starting a cycle. Patients who have had any dose reduction will not dose escalate. Selection of the time point for dose escalation in patients tolerating ixazomib was based the time course of treatment discontinuation due to AEs in previous ixazomib clinical studies in MM. A review of aggregate data from 275 patients participating in five phase 1/2 ixazomib MM studies (data cut-off, February 15, 2013) found 31 (12 %) patients who had discontinued due to AEs [7–10, 17]. Of these 31 patients, 27 (87 %) had discontinued by the end of cycle 4. This finding provides the rationale for dose escalation after 4 cycles in patients who are tolerating treatment. The study design for the phase 3 maintenance study, including the planned dosing schedule, is presented in Fig. 3. Adaptive dosing protocols that include provisions for individualized intra-patient titration based on safety and tolerability have informed dose-modification strategies for many recently approved oncology drugs, an approach that is considered useful in determining the optimal benefit–risk balance, particularly when long-term treatment to enable durable disease control is important [26, 36, 37]. This approach can maximize the proportion of patients who achieve bioactive exposures without undue risk for excessive toxicity [26].

The particular phase 1 study used for this analysis was selected because complete PK and efficacy/safety data were available for an adequately sized population of patients with MM who had been treated with single agent oral ixazomib. However, as the phase 1 population had relapsed/refractory MM and the phase 3 population will have newly diagnosed MM, there is a potential for some differences in response and tolerance. Patients with relapsed and/or refractory MM generally have significant tumor burden, whereas patients after ASCT and those in remission are likely to have less or no tumor burden, which may affect both the efficacy and toxicity of ixazomib. For example, interpretation of the exposure–safety analysis is based on the assumption that patients on the maintenance trial will have a similar risk of AEs to patients in the phase 1 study. However, patients enrolled in the phase 1study (*N* = 60) had received a median of 6 prior regimens over a median of 4.9 years since MM diagnosis [6], whereas patients receiving ixazomib in the maintenance setting following transplant will have received fewer prior therapies, which could result in a reduced risk of AEs, particularly a reduced risk of hematologic toxicities associated with less compromise of the bone marrow reserve.

To ensure that learnings from this population can be applied to the phase 3 maintenance study, non-hematologic AEs were categorized into two groups (≥grade 2 versus ≥grade 1) because patients on maintenance therapy will receive ixazomib for a long duration, at a time when they have asymptomatic MM and are therefore expected to have reasonable QoL with tolerable drug side effects. Under these conditions, even grade 2 toxicities (particularly non-hematologic toxicities) may have a significant impact on QoL. In addition, efficacy data were categorized into ≥SD versus PD because SD was considered to represent a therapeutic drug effect in this heavily treated relapsed and/or refractory MM population.

In conclusion, these exposure–response analyses indicate that a favorable benefit/risk balance may be achieved at ixazomib doses of 3 mg and 4 mg weekly, below the previously established MTD of 5.5 mg [6]. In the planned phase 3 maintenance trial, patients with newly diagnosed MM who have responded to induction therapy and HDT-ASCT will initiate ixazomib at a once-weekly dose of 3 mg, increasing to 4 mg if acceptable tolerability is established after 4 cycles, to provide maximum clinical benefit.
